# Supplementary figures and images for: Chitosan-Decorated Copper Oxide Nanocomposite: Investigation of Its Antifungal Activity against Tomato Gray Mold Caused by Botrytis cinerea
Source: Polymers (Basel). 2023 Feb 22;15(5):1099. doi: 10.3390/polym15051099 (PMC10007424; doi:10.3390/polym15051099)

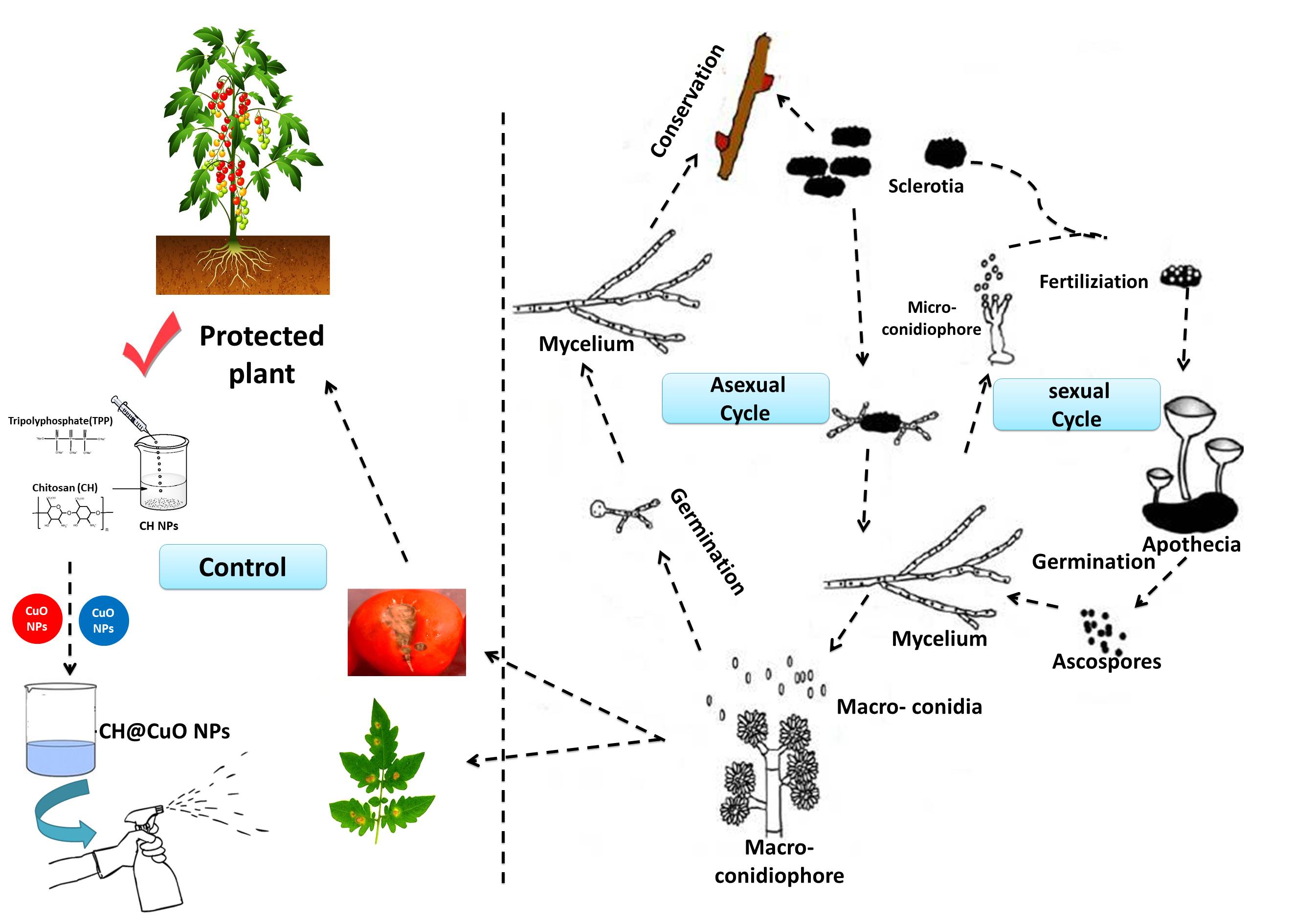


**Figure S1.** A diagram illustrating the characterization and control of *B. cinerea*

Supplement: Supplementary file 1 [file polymers-15-01099-s001.zip › polymers-2209827-supplementary.docx]
